# Modulation of Methacrylated Hyaluronic Acid Hydrogels Enables Their Use as 3D Cultured Model

**DOI:** 10.3390/gels9100801

**Published:** 2023-10-05

**Authors:** Ornella Ursini, Maddalena Grieco, Carla Sappino, Agostina Lina Capodilupo, Sara Maria Giannitelli, Emanuele Mauri, Alessio Bucciarelli, Chiara Coricciati, Valeria de Turris, Giuseppe Gigli, Lorenzo Moroni, Barbara Cortese

**Affiliations:** 1 National Research Council-Institute of Nanotechnology (CNR Nanotec), c/o Edificio Fermi, University Sapienza, Pz.le Aldo Moro 5, 00185 Rome, Italy; 2 National Research Council-Institute of Nanotechnology (CNR Nanotec), c/o Ecotekne, University of Salento, Via Monteroni, 73100 Lecce, Italy; maddalena.grieco@nanotec.cnr.it (M.G.); agostina.capodilupo@nanotec.cnr.it (A.L.C.); alessio.bucciarelli@nanotec.cnr.it (A.B.); chiara.coricciati@nanotec.cnr.it (C.C.); giuseppe.gigli@unisalento.it (G.G.); l.moroni@maastrichtuniversity.nl (L.M.); 3 Department of Chemistry, Sapienza University Rome, Pz.le A. Moro 5, 00185 Rome, Italy; carla.sappino@uniroma1.it; 4 Department of Science and Technology for Sustainable Development and One Health, Università Campus Bio-Medico di Roma, 00128 Rome, Italy; s.giannitelli@unicampus.it; 5 Department of Engineering, Università Campus Bio-Medico di Roma, 00128 Rome, Italy; emanuele.mauri@polimi.it; 6 Department of Chemistry, Materials and Chemical Engineering “G. Natta”, Politecnico di Milano, Piazza Leonardo da Vinci 32, 20133 Milan, Italy; 7 Department of Mathematics and Physics “Ennio De Giorgi”, University of Salento, Via Arnesano, 73100 Lecce, Italy; 8 Center for Life Nano- & Neuro- Science Italian Institute of Technology (IIT), 00161 Rome, Italy; valeria.deturris@iit.it; 9 Department of Complex Tissue Regeneration, MERLN Institute for Technology-Inspired Regenerative Medicine, Maastricht University, 6200 MD Maastricht, The Netherlands

**Keywords:** hyaluronic acid methacrylate, hydrogel, glioblastoma, tumour microenvironment

## Abstract

Bioengineered hydrogels represent physiologically relevant platforms for cell behaviour studies in the tissue engineering and regenerative medicine fields, as well as in in vitro disease models. Hyaluronic acid (HA) is an ideal platform since it is a natural biocompatible polymer that is widely used to study cellular crosstalk, cell adhesion and cell proliferation, and is one of the major components of the extracellular matrix (ECM). We synthesised chemically modified HA with photo-crosslinkable methacrylated groups (HA-MA) in aqueous solutions and in strictly monitored pH and temperature conditions to obtain hydrogels with controlled bulk properties. The physical and chemical properties of the different HA-MA hydrogels were investigated via rheological studies, mechanical testing and scanning electron microscopy (SEM) imaging, which allowed us to determine the optimal biomechanical properties and develop a biocompatible scaffold. The morphological evolution processes and proliferation rates of glioblastoma cells (U251-MG) cultured on HA-MA surfaces were evaluated by comparing 2D structures with 3D structures, showing that the change in dimensionality impacted cell functions and interactions. The cell viability assays and evaluation of mitochondrial metabolism showed that the hydrogels did not interfere with cell survival. In addition, morphological studies provided evidence of cell–matrix interactions that promoted cell budding from the spheroids and the invasiveness in the surrounding environment.

## 1. Introduction

Scaffolding materials that can biologically mimic the native microenvironment remain an open challenge in terms of both biocompatibility and control over the biomechanical and biochemical properties [1,2,3]. Three-dimensional in vitro models capable of replicating the physical and chemical complex tumour microenvironment has led to the investigation of a wide range of natural biomaterials (e.g., polysaccharides, such as alginate; hyaluronic acid (HA); or proteins, such as collagen, gelatin, fibronectin, laminin and fibroin), semi-synthetic materials (GelMA), synthetic materials (as PEG and self-assembled peptides) and tissues/organs derived decellularised extracellular matrix (dECM) (e.g., Matrigel and Geltrex) [4,5,6,7]. Although promising, these models present several drawbacks that limit their applications. For example, collagen and Matrigel-based scaffolds are limited in the tunability of their mechanical and chemical properties by the concentration of solution or require additional components. Furthermore, Matrigel is derived from murine tumours, which hinders its clinical use [8]. Fibrin gels lack mechanical stability or have suboptimal durability. Moreover, GelMA exhibits low viscosity with relatively fragile properties and fast degradation, as well as not being able to polymerise with sufficient rapidity at body temperature, which can affect gel–cell confinement [9,10]. On the other hand, synthetic hydrogels show improved mechanical properties but lack the biological properties and compatibility of ECM-based materials, and require modification to elicit cell-specific adhesion [11]. Moreover, most of these models do not employ HA, which is a significant component of the brain ECM [12,13]. In fact, the HA family is present in the brain microenvironment at high levels with different molecular weights (MWs) (high MW > 1000 kDa, low MW 20–1000 kDa and oligosaccharides ~0.4–20 kDa) and accumulates in response to insults, such as injury, inflammation and infection, or correlates with cancer, such as glioblastoma (GBM) [14,15,16,17]. Meanwhile, lower amounts of other fibrous proteins (i.e., collagen, fibronectin and vitronectin) and basement membrane proteins (i.e., laminin) are mainly restricted to the vascular and perivascular spaces in the brain [18,19,20]. Thus, biomaterial studies that modelled the brain ECM increasingly converged on HA hydrogels showing that the concentration, degree of chemical modification, matrix stiffness and porosity can affect the physiochemical properties of the hydrogel, as well as cell behaviour. Methacrylation of HA (HA-MA) was shown to improve the resistance to enzymatic degradation compared with unmodified HA [21], as well as being ideal in targeting tumours for drug delivery applications in cancer therapy [22]. Moreover, an increasing amount of literature has described that the chemical modification with methacrylate groups aids improve the mechanical and physical properties of the HA via changes in the degree of methacrylation (DM) or in the MW, as well as by varying the ultraviolet (UV) intensity and exposure time. However, studies reported till now show great heterogeneity in the HA-MA physical properties, procedures of synthesis and polymerisation processes used, which do not allow for easy control of the substitution degree of the reaction product and a definite reproducibility of synthesis. For example, Tsanaktsidou and her team investigated HA-MA hydrogel systems by comparing both high and low HA MWs and DMs [23]. They showed that the MW of HA or/and DM of HA-MA affected the hydrogel’s properties and rheological profile. Burdick and colleagues showed that networks of HA-MA hydrogels can be modulated by varying their concentration, the number of reactive groups and the MW of HA [24]. Oudshoorn reported that HA-MA with higher DM became progressively stiffer due to the higher network density, showing that the DM affected the compressive modulus [25]. Other studies highlighted the impact of HA-MA in vitro hydrogels on the cell–matrix interactions in both primary and secondary brain cancers [14,15,26,27,28]. In particular, Pibuel and colleagues reported that both high and low MWs of HA enhanced the migration of GBM cells without affecting their viability [14]. Chen and co-workers used hydrogels with similar composition and the same concentration of HA-MA but different MWs to study the effect of the chain length of HA crosslinked with the GelMA network on the invasive behaviour of patient-derived xenograft cells. They reported a reduced invasive behaviour with respect to cells cultured in lower MW hydrogels [15]. Yet, the same authors reported in a separate study that a low MW HA did not affect the metabolic activity of U251-MG cells, showing a reduced invasiveness [28]. Despite the controversial results on HA contribution, its presence still is clearly significant in many signalling pathways and the crosstalk of GBM invasiveness. Besides the biochemicals aspects, HA was also shown to biophysically and dimensionally impact the crosstalk of GBM progression, proliferation and survival [26]. For example, Ondeck reported on the modulation of the mechanical properties of HA-MA hydrogels by monomer concentration, duration of UV exposure and DM [29]. Moreover, a high MW HA can also influence the strengthening of the 3D polymer network and its binding with cellular receptors, such as CD44 and RHAMM (receptor for hyaluronan-mediated motility) [30,31,32]. This was likely caused by a major affinity of binding due to longer HA chains, as well as enhanced receptor clustering and HA internalisation into cells [33,34]. Camci-Unal and colleagues observed an increase in cell spreading with the hydrogel stiffness in 2D, whereas in 3D, it was correlated with larger pore sizes and lower stiffnesses [35]. Wang developed HA-MA with different DMs, showing that a DM of around 60% exhibited optimal porosity, enhanced cell adhesion, and increased invasive capacities and malignancy in a 3D system in comparison with 2D monolayer cultures [36]. Thus, for all the abovementioned reasons, there is a strong need for the improved characterisation of changes in matrix stiffness to develop hydrogel platforms amenable to studies of GBM tumour invasion.

In this work, we present the investigation of synthetic parameters of photo-crosslinkable HA-MA in an aqueous medium to control the DM of the polymer using HA with a high MW (Scheme S1). Thus, we optimised the photopolymerisation process in relation to the chemical nature and concentration of the photoinitiator and the irradiation energy to obtain a suitable in vitro hydrogel platform. The mechanical properties of the hydrogel constructs, which were obtained by first varying the concentration of the photoinitiator Irgacure 2959, were characterised to determine differences between hydrogels with different DM. Moreover, as the choice and optimisation of the photoinitiator remain crucial in the control of the physiochemical features for any 3D platform, we compared the characteristics of hydrogels crosslinked with the two most widely studied photoinitiators (Irgacure 2959 and LAP) under the two different wavelengths. The rheological analyses and the mechanical properties studies allowed us to determine the best platform with the most similar brain-mimetic ECM characteristics. Preliminary in vitro cell culture studies using human glioblastoma cells (U251-MG) revealed that our optimised hydrogels were efficient for cell viability, metabolic activity, cell proliferation and cell morphology in both 2D and 3D dimensionality. We showed that our hydrogels supported glioblastoma cell growth and behaviour better within 3D platforms, showing distinct responses compared to 2D platforms and an environment mimicking the native tissue architecture. Our results highlight a reliable synthetic procedure, producing manageable hydrogels with stable mechanical properties suitable for clinical applications. With the ability to precisely manipulate the microenvironment, our optimised hydrogels open new avenues for exploring novel therapeutic strategies and addressing critical challenges in glioblastoma treatment and beyond.

## 2. Results and Discussion

### 2.1. HA-MA Stiffness Varied with Photopolymerisation Parameters and DMs

Undoubtedly, one of the major challenges in biomaterial science is the achievement of highly reproducible and comparable HA-MAs given the different methods of synthesis. Conjugation of the methacrylate group to HA chains was reported using methacrylic anhydride [37,38,39,40,41] or glycidyl methacrylate [25,42,43,44]. Methacrylation with glycidyl methacrylate is an alkylation reaction that allows for a high DM but suffers from several drawbacks, such as a large excess of glycidyl methacrylate and strong basic conditions, the presence of organic solvents and long reaction times. Instead, HA-MA synthesis using methacrylic anhydride is a simpler esterification reaction that can be carried out in aqueous conditions [24,45]. The methacrylation reaction of HA chains allows for the introduction of photo-crosslinkable reactive groups, which enable the formation of crosslinked polymers with different bulk properties [42,46]. Thus, we introduced methacrylate functions on the HA polymeric chain under mild conditions and in aqueous medium, modulating the experimental conditions [37,38,40].

To ensure the success of the reaction, HA-MA polymers were characterised using Fourier-transform infrared (FT-IR) spectroscopy and ^1^H-NMR spectroscopy (Supplementary Information, Figures S1 and S2), which confirmed the methacrylation of the HA backbone and allowed for a DM assessment. As the DM is of paramount importance in the crosslinking density since it affects the mechanical properties and porosity, we first compared the physicochemical properties of different DMs, specifically with high methacrylation of ~98.0% (HM) and low methacrylation of 24.7% (LM). Among the photoinitiators conventionally exploited to photocrosslink hydrogels, the most predominantly used is the free radical type I photoinitiator Irgacure 2959. This photoinitiator normally requires high concentrations for an efficient crosslinking process (studies reported concentrations up to 0.5% to not compromise cell viability) [41,42,47]. The result of the crosslinking process is also correlated with the length of UV exposure. Therefore, minimizing the concentration of the photoinitiator and light intensity aids in reducing cell toxicity but at the expense of the longer crosslinking times (10–60 min) that are necessary to achieve adequate biomechanical properties [48,49]. Also reducing the crosslinking time of HA-based materials is necessary for in situ polymerisation in many biomedical applications. Thus, hydrogels were initially polymerised using Irgacure 2959 (with concentrations ranging from 0.1% or 0.3% *w*/*v*) to evaluate the modulation of the mechanical properties of HA-MA hydrogels synthesised with different DMs and wavelengths. Indeed, the absorbance of the Irgacure 2959 drops rapidly above 300 nm [50] such that shorter wavelength UV sources crosslink the hydrogel more efficiently. As a result, different light sources with different wavelengths were exploited.

**Figure 1:**
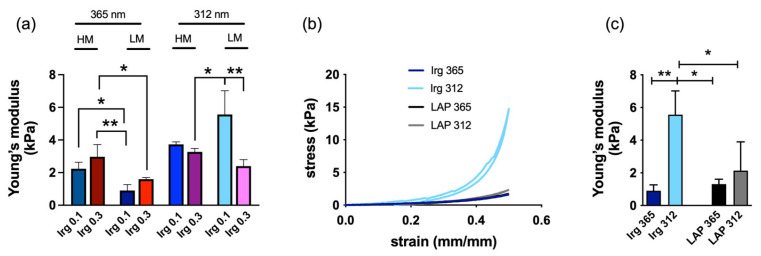
(**a**) Comparison of the Young’s modulus of hydrogels with high methacrylation (~98.0%, referred to as HM) and low methacrylation (24.7% referred to as LM), with different concentrations of the photoinitiator (Irgacure, referred to as Irg), and exposed to the different wavelengths 365 nm and 312 nm. (**b**) Compressive stress–strain curve of hydrogels with different photoinitiators and exposed at different wavelengths. (**c**) Comparison of the Young’s modulus of LM hydrogels with different photoinitiators and exposed to the different wavelengths 365 nm and 312 nm. Data were from 3 independent experiments. Error bars represent ± S.D. Significance was determined using one-way and two-way ANOVA. * *p* < 0.05, ** *p* < 0.01.

The Young’s modulus analysis showed that regardless of the DM, at a wavelength of 365 nm, the material stiffness increased with the photoinitiator concentration (Figure 1a). Yet, an exposure at a wavelength of 312 nm showed a tendency of decreased hydrogel stiffness with an increase in the photoinitiator concentration. Hydrogels photopolymerised at the same concentrations and the same DM showed a higher stiffness when exposed to a wavelength of 312 nm with respect to those photopolymerised at 365 nm. An increase in the concentration of photoinitiator supported the formation of free radicals, which, in turn, depended on light intensity, resulting in a more loosely crosslinked structure with higher wavelengths. However, one limiting factor in increasing the amount of photoinitiator is its cytotoxic effects, which are detrimental to cells due to the presence of free radicals in the photoinitiator. Toxicity can be reduced by minimizing the photoinitiator concentrations, but this also can invalidate the efficiency of the crosslinking of the hydrogel or using alternative photoinitiators that exhibit enhanced efficiency and biocompatibility, such as lithium phenyl-2,4,6-trimethylbenzoylphosphinate (LAP) [51,52]. However, the selection of the photoinitiator might also influence the physiochemical and biological effects of the hydrogels.

Furthermore, although higher DM values are preferred for photo-crosslinked hydrogels developed in situ, increased crosslinking densities generally yield denser matrices and tighter networks [42]. Thus, to obtain less stiff hydrogels, which could match better the stiffness of the brain, we decided to use only the HA-MA polymer with a DM of 24.6% and investigate the physical properties as a function of the two UV irradiances of 312 and 365 nm.

The mechanical properties determined under compression stress showed the highest value for hydrogels cured with Irgacure at 312 nm with respect to hydrogels cured with Irgacure at 365 nm and hydrogels cured with LAP (Figure 1b). In particular, the compressive stress of hydrogels cured with LAP at 365 nm and 312 nm showed overlapping curves. These results expressed the hydrogels’ good reversibility and preservation of its elastic nature, showing good shape recovery behaviour, even undergoing 50% deformation of its original height.

Young’s modulus (E) as a measure for the stiffness of an elastically deformable material, confirmed a compressive modulus of hydrogels exposed to a wavelength of 312 nm, higher than those exposed to 365 nm (Figure 1c). Specifically, Young’s moduli of hydrogels cured with Irgacure and exposed to a wavelength of 312 nm (E ≃ 5.5 ± 1.4 kPa) were significantly higher compared with those exposed to a 365 nm wavelength (E ≃ 0.9 ± 0.3 kPa), while hydrogels crosslinked with LAP and exposed to a wavelength of 312 nm showed an E ≃ 2.1 ±1.7 kPa, slightly higher with respect to those exposed to a 365 nm wavelength (E ≃ 1.3 ± 0.3 kPa).

Our results indicate that the stiffness variation between the hydrogels was highly dependent on the photocatalyst nature and UV wavelength, independent of the DM.

### 2.2. Different Photoinitiators and UV Crosslinking Affected the Rheological Parameters of the Hydrogel

**Figure 2:**
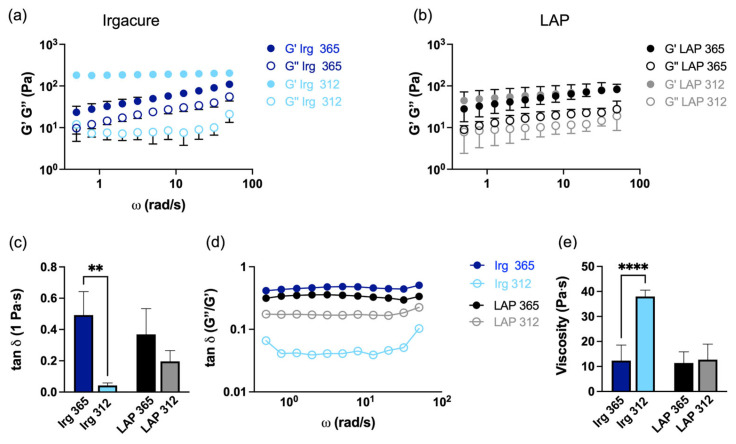
Effect of different photoinitiators and wavelengths on the rheological properties of HA-MA (DM 24.6%). Frequency sweep of hydrogels crosslinked with (**a**) Irgacure (referred to as Irg) 0.1% and (**b**) LAP exposed to the different wavelengths of 365 nm and 312 nm. (**c**) tan δ (1 Pa·s) of hydrogels and (**d**) frequency sweep of tan δ for hydrogels. (**e**) Viscosity (1 Pa·s) properties of all hydrogels. The data are presented as the mean ± SD of three independent experiments. * indicates statistically significant difference using one-way ANOVA with ** *p* < 0.01 and **** *p* < 0.0001.

The rheological properties of the hydrogels were measured to compare the different photoinitiators and wavelengths. To investigate the time-dependent behaviour of the hydrogels in the non-destructive deformation range, frequency sweep tests were undertaken (Figure 2a,b). Different values of the storage modulus (G′) and loss moduli (G″) were obtained for all hydrogels, hinting at a clear dependence of the rheological properties as a function of the different photoinitiators and wavelengths. In particular, hydrogels exposed to a 312 nm wavelength presented an almost constant G′, which is an indication of good stability of the crosslinked network (Figure 2a,b). Moreover, the G′ of hydrogels obtained using Irgacure and exposed to a 312 nm wavelength was higher than those of hydrogels with LAP exposed at the same wavelength, while the G′ of all hydrogels exposed to 365 nm showed comparable values (Figure S3a). Thus, the exposure of hydrogels to a 312 nm wavelength led to higher values of G′ and lower values of G″ with respect to samples exposed to 365 nm, independent of the photoinitiator used (Figure 2a,b), which shows good stability of the crosslinked network. Comparing the storage moduli values of our hydrogels to those of tissues of the brain and nerves [53,54,55] (Figure S3c) showed that these hydrogels were within this range (~100–150 Pa), as well as including important bioactive features of the brain ECM, such as the presence of HA, and thus, are ideal to be used as scaffolds for brain tumour microenvironments.

Strain sweep test (amplitude sweep) results were characterised by a storage modulus G′ larger than the loss modulus G″, regardless of the wavelength to which they were exposed or the photoinitiator used (Figure S4a,b). This suggested that all hydrogels displayed a predominantly solid-like behaviour rather than a fluid-like state [56]. The relative contribution of the viscous components to the mechanical properties of the material was evaluated through the loss factor (tan δ), which was calculated by taking the ratio between G″ and G′ from the rheological measurements. This parameter gives an indication of the proportion of dissipated energy to stored energy. Specifically, values of tan δ < 0.1 are indicative of a hydrogel that is predominantly elastic, which stores the energy rather than dissipating it during deformation. Instead, hydrogels that exhibit a higher tan δ, exhibit a better absorption and dissipation of energy [57]. Moreover, tan δ specifies whether a gel is strong (tan δ < 0.1) or weak (tan δ > 0.1) [58]. We noticed that only hydrogels crosslinked with Irgacure at 312 nm corresponded to strong gel behaviour (Figure 2c,d), whereas the other hydrogels were related to weak gel behaviour. These results corroborated the findings of our mechanical measurements. More specifically, Figure 2d shows that all hydrogels reported a tan δ that was mostly independent of the frequency, confirming that all systems were primarily elastic. In particular, hydrogels crosslinked at 365 nm showed a higher tan δ, whereas the tan δ values of hydrogels crosslinked at 312 nm were contingent on the photoinitiator used (showing higher values only for LAP). Moreover, only platforms cured with Irgacure at 312 nm showed a tan δ that increased with the lowest and highest frequencies. The viscosities of the hydrogels, which were calculated at 1 Hz and reported in Figure 2e, showed that HA-MA hydrogels crosslinked with 1% Irgacure were significantly (*p* < 0.0001) more viscous when exposed to 312 nm (37.9 ± 2 Pa·s) than to 365 nm (11.8 ± 1.7 Pa·s), whereas hydrogels crosslinked with LAP were less viscous with comparable values (11.5 ± 3 Pa·s for a 365 nm wavelength exposure, 12.7 ± 7 Pa·s for a 312 nm wavelength exposure). These results are in accordance with the result of the elastic modulus obtained from the stress–strain curve.

### 2.3. Morphology of the Hydrogels Varied with Photoinitiators and UV Wavelength

**Figure 3:**
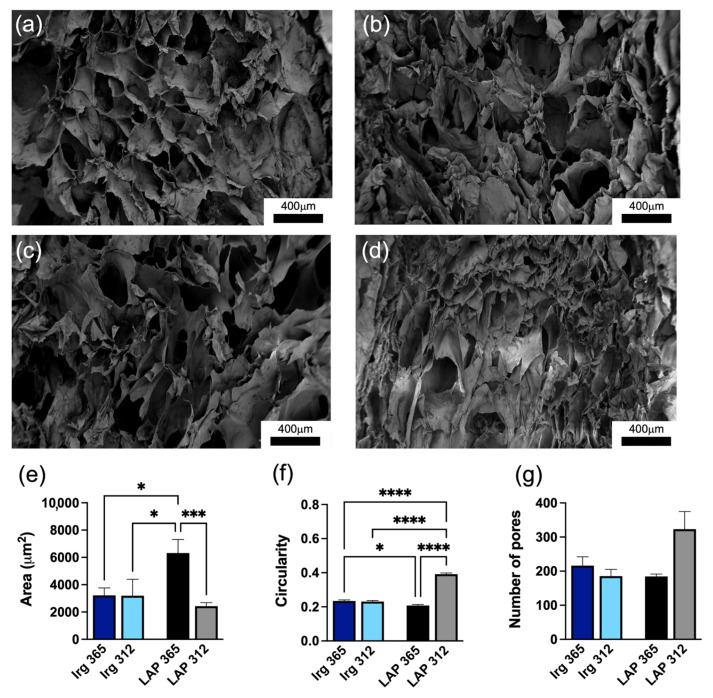
Representative SEM images of cross-section of the hydrogels crosslinked with (**a**) Irgacure 0.1% at 365 nm, (**b**) Irgacure 0.1% at 312 nm, (**c**) LAP at 365 nm and (**d**) LAP at 312 nm. Scale bar: 400 mm. Quantification of the morphological features of the pores, showing (**e**) pore size area, (**f**) circularity and (**g**) number of pores. Error bars represent the s.e.m. of three independent fields of acquisition. * indicates statistically significant difference using one-way ANOVA with * *p* < 0.05, *** *p* < 0.001 and **** *p* < 0.0001.

To verify whether the photopolymerisation process impacted the pore structure, scanning electron microscopy (SEM) analyses were carried out. Figure 3a–d display the SEM micrographs of the hydrogels with the two photoinitiators and different wavelengths.

Low magnification (100×) images revealed relevant information about the pore geometry, apparent pore size and heterogeneity of the overall hydrogel networks. All hydrogels showed pores with elongated irregular shapes, as the aspect ratio was higher than 1.00 (Figure S5a). Hydrogels photopolymerised with Irgacure showed that the change in wavelength did not influence the pore structure, specifically area (Figure 3e), circularity of the pores (Figure 3f) or aspect ratio (Figure S5a). Hydrogels photopolymerised with LAP were found to be more heterogeneous and influenced by the wavelength used. In particular, hydrogels crosslinked with LAP at 365 nm showed bigger pores (Figure 3e) with larger values of Feret diameter (Figure S5b), whereas at 312 nm, the hydrogels exhibited a higher number of pores (Figure 3g), also exhibiting the most elongated shape (largest aspect ratio value) (Figure S5a).

Larger pores are usually present in less dense hydrogels where fewer crosslinks are generated [59]. Our results confirmed the results of previously reported work, which showed that the stiffness of the hydrogels is inversely proportional to their pore sizes [60].

It is important to underline that the values obtained from the SEM images represent the morphology of lyophilised hydrogels to highlight differences between the samples exposed to different wavelengths. To summarise, the wavelength and photoinitiator modulated the microstructure of the hydrogel system.

### 2.4. HA-MA Hydrogel Promoted Cell Survival

As the stiffness and porosity of the gel structure play important roles in dictating the cell behaviour and invasive capacity of GBM cells in HA-containing hydrogels [28,61,62], our results verified that hydrogels crosslinked with LAP and exposed to a 312 nm wavelength better represented an ECM biomimetic environment for glioblastoma investigations. Moreover, platforms that are less crosslinked are more favourable for cell viability due to lower constraints in nutrient transport through the network [24].

Thus, we assessed preliminary in vitro tests using hydrogels crosslinked with LAP at 312 nm. The capacity of a hydrogel to absorb nutrients is responsible for subsequent cell adhesion, diffusion and proliferation [63]. For this reason, we first evaluated the protein absorption (Figure S5). As shown, 70% of an amount of 1 mg/mL of BSA with respect to the control in water was adsorbed by HA-MA hydrogels, which indicates an improved protein absorption capacity. This was attributed to the intermolecular hydrogen bonding between the hydroxyl groups of the HA-MA hydrogels and the BSA [64].

**Figure 4:**
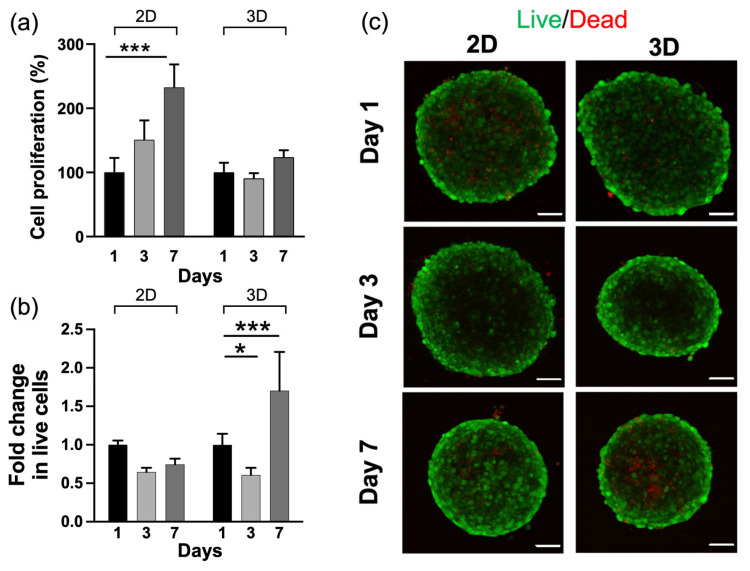
Biocompatibility of U251-MG spheroids generated using the hanging drop array method seeded on the hydrogel in different microenvironments. (**a**) Metabolic activity of U251-MG spheroids after 1, 3 and 7 days cultured on 2D HA-MA and embedded in 3D HA-MA. Cell viability (live/dead assay) of U251-MG spheroids, in the 2D and 3D microenvironments with (**b**) normalised fold change of live cells over time compared with day 1 and (**c**) representative confocal image of U251-MG spheroids, where all cells were counterstained with calcein AM (green) and dead cells were marked with propidium iodide (red). Scale bar indicates 100 μm. The results are shown as percent viability relative to day 1, which was considered to be 100%. The values are the mean ± s.e.m. of three independent experiments. * indicates statistically significant difference using one-way and two-way ANOVA with * *p* < 0.05 and *** *p* < 0.001.

As cell sensing, proliferation and invasiveness are influenced by the dimensionality of the microenvironment [65,66,67], we, thus, compared 2D systems, with the cells cultured on top of the hydrogels, with 3D systems, with the cells within the hydrogels. We first profiled the metabolic activity and viability of cells for 1, 3 and 7 days, seeding U251-MG spheroids on 2D and 3D HA-MA microenvironments (Figure 4a). The percent of metabolic activity of cells was measured by comparing the MTT absorbance for hydrogels in 2D and 3D and showed an increase up to 7 days in the 2D platform, indicating an increase in energy metabolism, as shown in Figure 4a. On the other hand, in the 3D platform, cells did not show any modulation in succinate dehydrogenase activity, indicating stability of energy metabolism over the time course. As cytocompatibility is a crucial evaluation for biomaterials, U251-MG spheroids were also imaged using a live/dead assay to assess the survival and distribution of U251-MG spheroids within the hydrogels using confocal microscopy for 1, 3 and 7 days. Calcein-AM-stained viable cells characterised mostly the bulk of the spheroid, while dead cells were negligible in the spheroids. Neither 2D nor 3D microenvironments evinced noticeable cytotoxicity, indicating satisfactory cytocompatibility of the hydrogels (Figure 4b). The increase in metabolic activity up to 7 days in the 2D platform was hypothesised to be related to a reduced contact surface area with the hydrogel, resulting in an over confluence, leading to contact inhibition of growth [68,69,70], which was also confirmed by the live/dead analysis. In fact, it was shown that contact inhibition can induce a high metabolic rate [71]. On the other hand, the metabolic activity in 3D compared with 2D was stable over time, despite a slight reduction in live cells at 48 h. On day 7, the cell viability in the 3D settings showed an increase, although there was the presence of a small necrotic core reminiscent of a physiological tumour environment, which can develop due to the lower diffusion of nutrients and oxygen, leading to hypoxic conditions [72]. The increase in the contact surface area is also a key point in favour of a 3D system and encourages the hypothesis of a system that is able to promote the cellular spread typical of aggressive tumours, supporting the morphological analyses performed to assess the spheroid growth invasiveness [73,74].

### 2.5. Morphological Changes of Tumour Spheroids on HA-MA Hydrogel

**Figure 5:**
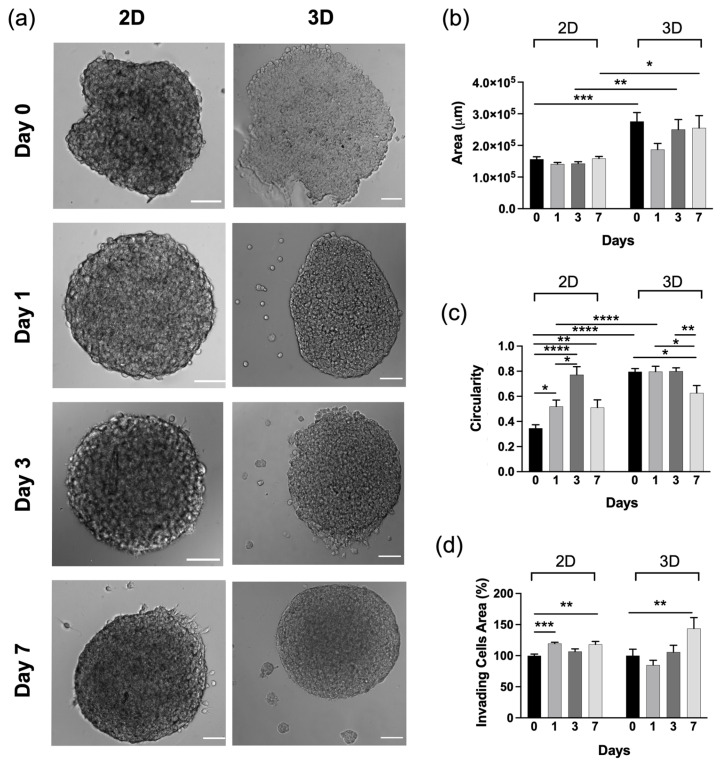
(**a**) Representative phase contrast micrographs of U251-MG spheroids in 2D and 3D microenvironments at different time points (days 0, 1, 3, 7). Scale bar: 100 μm. (**b**,**c**) Bar graph results of morphological characterisation of spheroids in the different microenvironments showing (**b**) spheroid area and (**c**) circularity. (**d**) Invasive capacity over time of U251-MG, showing differences in invasive capacity across microenvironments. The values are the mean ± s.e.m. of three independent experiments. * indicates statistically significant difference using one-way and two-way ANOVA with * *p* < 0.05, ** *p* < 0.01, *** *p* < 0.001 and **** *p* < 0.0001.

As cell invasiveness is a critical function in the metastatic cascade, we next analysed time-lapse images of the spheroids in the two different microenvironments. Figure 5 shows the morphologies of spheroids after 1, 3 and 7 days in both microenvironments. Initially, the spheroids were quite polydispersed. This was attributed to the method used to obtain the spheroids (the hanging drop method), which usually does not seem to yield very round spheroids [75].

**Figure 6:**
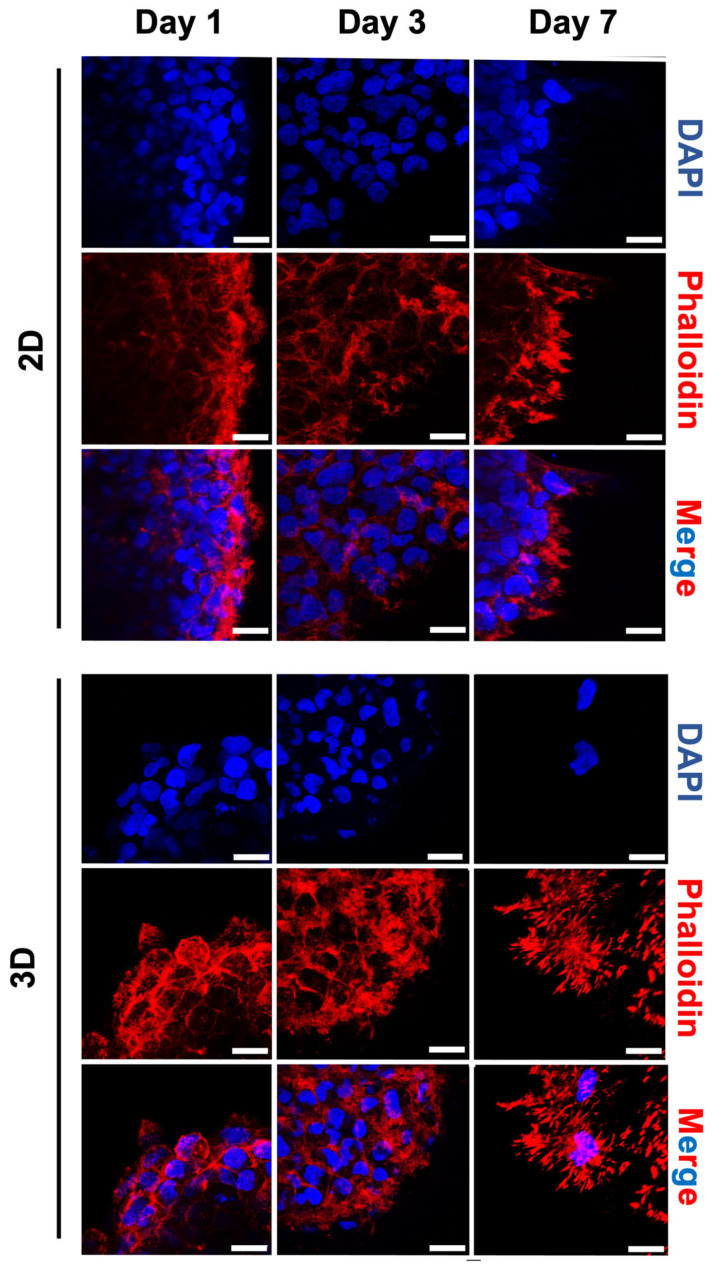
Representative confocal images showing cytoskeleton and nuclei staining with Phalloidin and DAPI for the U251-MG spheroids cells that were encapsulated in 2D (**top**) and 3D (**bottom**) hydrogels. Fluorescent images were captured on days 1, 3 and 7. Scale bar: 50 μm.

The spheroid’s surface area in 2D did not show a significant size increase but they initially increased their spherical shape or circularity (up to 3 days), which was related to the cell proliferation and cellular reorganisation due to the partial interaction of the spheroid system with the surrounding surface. Spheroids in the 3D platform seemed to adapt more to the microenvironment by increasing their area, probably due to higher cell–matrix interactions than in the 2D platform (Figure 5b). Furthermore, the shape of the spheroid in 3D did not initially (up to 3 days) change significantly, which was probably related to the more confined hydrogel environment. The 2D and 3D microenvironments on day 7 showed a slight decrease in circularity, which could be related to the invasive properties of cells (Figure 5c,d). In fact, spheroids in 2D hydrogels showed a slow increase in invading cell area at the spheroid edge (Figure 5d). In 3D, instead, the spheroids showed a higher increased invasion capacity into the surrounding gel. Differences in the spheroid’s invasive area may have been due to the microstructure difference between 2D and 3D, as the pores’ topography of the 3D microenvironment may facilitate the migration of cells. This was associated with the fact that in 3D, spheroids are encapsulated within the hydrogel, while in 2D only a segment of the spheroid can interact with the substrate. In addition, we observed the cytoskeletal arrangement in 2D and 3D cell culture hydrogels after staining the U251-MG spheroids with F-actin (Figure 6).

Our results complement previous findings that demonstrated how HA effectively links the actin cytoskeleton to the local ECM [73]. In fact, cells on the 2D and 3D HA-MA hydrogels initially displayed fewer spreading protrusions, as expected due to the early time point. After 3 days of culture, the spheroids showed minimal cytoskeleton spreading. Around day 7, spheroids in the 3D microenvironment showed remodelling of the matrix surrounding them, with cells found to progressively detach from the spheroid infiltrating via individual protrusions in a circular manner. This is in line with the fact that in a 3D microenvironment, cells involved in the migration process require reshaping of their cytoskeletal architecture with the formation of lamellipodia, filopodia and actin myosin contractility [76,77] to migrate through the pores of the hydrogel, as also happens in the openings of the natural ECM.

### 2.6. Glioblastoma Spheroids Showed a Proliferative Gradient on HA-MA Hydrogel

**Figure 7:**
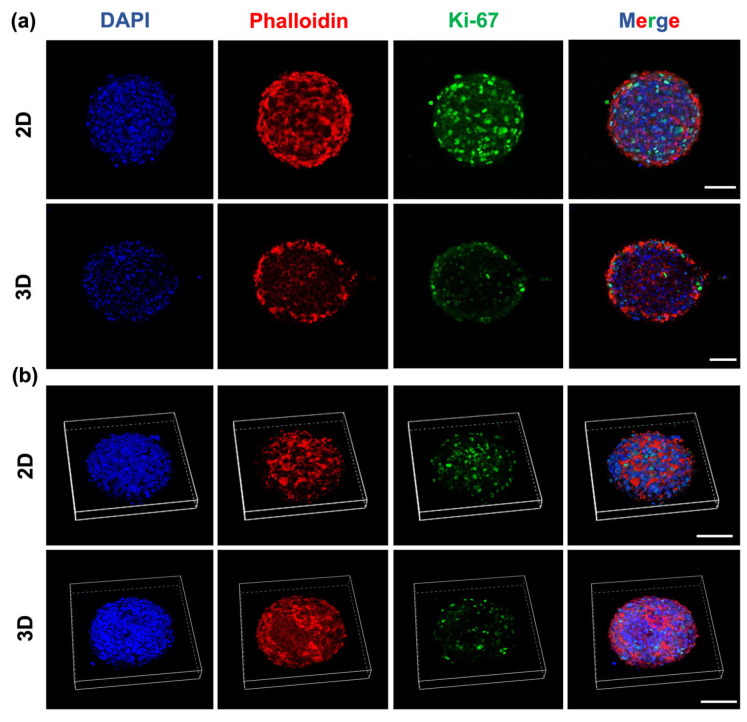
Representative (**a**) two-dimensional and (**b**) surface rendering confocal images showing the cell proliferation detected using Ki-67 of U251-MG spheroids in 2D and 3D microenvironments, where red is Phalloidin-TRITC, green is Ki-67 and blue is DAPI. Scale bar: 100 μm.

As cell proliferation depends on the invasion process, as well as the microenvironment in both 2D and 3D, we checked for the expression of Ki-67, which is a well-established cellular marker of proliferation, using immunofluorescence (Figure 7). As expected, the proliferation marker Ki-67 in the 2D cultures was observed in both the inner and outer regions of the spheroids. Conversely, in 3D, Ki-67 was detected mainly in the cells located on the leading edge in the outer region of the spheroids, but not in the inner region. Previous studies showed that the proliferation rates are different in 2D and 3D, reporting a reduced proliferation in 3D spheroid cultures [78] and hydrogels [79,80,81] with respect to 2D monolayer cultures.

Our qualitative analysis of Ki-67 corroborated these findings, as in the 3D microenvironment, the presence of the Ki-67-positive cells was visible, with more being located on the outer proliferating rim. The reduction in the proliferative capacity of the 3D settings was thus responsible for the fall in metabolic cell number. These results confirmed that proliferation contributed to the invasion process [82].

## 3. Conclusions

In this work, we synthesised and characterised HA-MA hydrogels by comparing photoinitiators and UV wavelengths to tailor a wide range of physical properties. We specifically chose to look at several key design relationships, such as the effects of the DM, nature and concentration of photoinitiator, as well as the UV intensity, on the hydrogels’ properties, keeping the MW of the HA constant. The physical properties of the hydrogels allowed us to narrow down the selection of parameters to obtain a reproducible, 3D and mechanically well-defined model that can depict the HA milieu of the brain ECM while reproducing its biophysical properties. Thus, the possibility to control the mechanical properties and porosity makes our hydrogels ideal as a biomimetic environment. Preliminary in vitro data showed the cytocompatibility of our hydrogels, exhibiting good viability of 2D and 3D systems. Insights from future studies using our hydrogel will facilitate mechanistic studies on how the microenvironmental conditions regulate GBM invasiveness and response to therapy. Upon embedding multicellular tumour spheroids, this biomimetic tumour environment provides a valuable new tool to meet the requirement of biomaterials applied in different fields of biomedical sciences.

## 4. Materials and Methods

### 4.1. Materials

HA sodium salt (MW 1500–2200 kDa) was purchased from Acros Organics. Methacrylic anhydride (MA), *N*,*N*-dimethylformamide (DMF), Irgacure 2959 and phosphate-buffered saline (PBS) were purchased from Sigma-Aldrich (Sigma-Aldrich, Steinheim, Germany). Lithium Phenyl-2,4,6-trimethylbenzoylphosphinate (LAP) was purchased from Tokyo Chemical Industry Co., (Tokyo, Japan). The dialysis membrane Spectra-Por 7 MCWO 10 kD was purchased from Spectrum Labs (San Francisco Bay Area, CA, USA).

### 4.2. Synthesis of Methacrylated Hyaluronic Acid (HA-MA)

Different molar ratios between HA and methacrylic anhydride (MA) in a mixture of water/DMF were used to synthesize HA-MA polymers, following a procedure described previously [38,40] with some modifications (Table S1), as discussed in the Supplementary Information. In brief, an amount of HA (ranging between 1.0–3.0 g) was dissolved in ultrapure water in order to obtain different concentrations of HA (between ~1 and 2% wt solution) under continuous stirring overnight until complete dissolution at room temperature. Subsequently, DMF was added dropwise to the solution in a 3:2 ratio (water:DMF) and the mixture was cooled down to 4 °C. MA was added slowly dropwise. The pH was carefully monitored to remain between 8 and 9 via the addition of 0.5 M or 0.1 M NaOH for 4 h. Then, the reaction was kept at 4 °C under continuous stirring for one night in the dark. Subsequently, the reaction was stopped by adding sodium chloride to the mixture, achieving a final concentration of 0.5 M NaCl.

The polymer was precipitated by the addition of very cold ethanol (2:3 in *v*/*v*, water:EtOH). After the removal of the supernatant, the precipitate was subsequently washed two times with mixtures of water:EtOH (3:7 in *v*/*v*) and once with water:EtOH (1:4 in *v*/*v*). The final precipitate was dissolved in ultrapure water and further purified using dialysis membrane (Spectra-Por MWCO 10 kDa) against ultrapure water for 72–96 h (water was changed every 8–10 h). The purified product was recovered via freeze-drying and characterised using ^1^H-NMR and FT-IR analyses. The synthetic details and results of the prepared HA-MA are summarised in Table S1 in the Supplementary Information.

### 4.3. Preparation of Hydrogels HA-MA

HA-MA solutions were subsequently prepared in PBS at 1% *w*/*v* with either 0.03% *w*/*v* LAP (Lithium Phenyl-2,4,6-trimethylbenzoylphosphinate) or 0.1% and 0.3% *w*/*v* Irgacure 2959 (1-[4-(2-hydroxyethoxy)-phenyl]-2-hydroxy-2-methyl-1-propanone). After the dissolution of the catalyst, the mixture was exposed to light irradiation using two different light sources: a UV lamp at 365 nm (UV intensity 0.7 mW/cm^2^) and a UV transilluminator at 312 nm (UV intensity 1.5 mW/cm^2^). All different conditions used to form HA-MA hydrogels are reported in Schemes S2 and S3 in the Supplementary Information.

### 4.4. Characterisation of Methacrylated HA Using ^1^H-NMR Spectroscopy

The DM was measured at room temperature using a proton nuclear magnetic resonance (^1^H-NMR) with a Bruker AC 300 (300 MHz) and a Bruker AVANCE III 400 MHz. The HA-MA polymer samples were dissolved in D_2_O. Spectra were measured by comparing the integral related to protons signal at 5.8 ppm or 6.2 ppm, which was assigned to the methylene protons of the methacrylate group protons (2 s, 2H, -CO-C(CH_3_)=CH_2_ belonging to MA residues linked to HA), with the integral related to protons in a range between δ = 1.88 ppm and 1.81 ppm (considering only the contribution of NH-CO-CH_3_ belonging to HA). Specifically, the DM was measured by comparing the integral related to the methyl group belonging to HA residues (δ = 1.88 ppm) with the integral of the signal of the CH_3_ belonging to MA (δ = 1.81 ppm) (Figure S1, Supplementary Information).

### 4.5. Characterisation of Methacrylated HA Using FT-IR Spectroscopy

The FT-IR spectra were collected using a Nicolet 8700 FT-IR spectrometer (Thermo Scientific) equipped with an attenuated total reflectance (ATR) sampling accessory with a single bounce diamond crystal. FT-IR analyses were used to corroborate the chemical structure and interactions of HA-MA compared with those of HA (Figure S2, Supplementary Information). New bands at 1706 cm^−1^ and 1300 cm^−1^ were ascribed to the stretching of C=O groups (ν_as_ COO at 1706 cm^−1^) and the scissoring of –C=C-H at 1300 cm^−1^ due to the introduction of methacrylate residues. The band at 946 cm^−1^, which was associated with the wagging of –C=C-H, was also present in the spectrum of pure HA due to the bending of the hydroxylic group, but its intensity increased when methacrylate residues were linked to HA. This behaviour is in agreement with previous data reported in the literature [37].

### 4.6. Mechanical Characterisation of Hydrogels

The bulk compressive moduli of hydrogels were determined by using a universal testing machine (model 3365, Instron Corporation, Issaquah, WA, USA) equipped with a 10 N load cell. Cylindrical samples (ϕ = 12 mm, h = 6 mm) were compressed at a crosshead speed of 5 mm/min up to 50% strain. A load/unload cycle was recorded for each sample. The compressive modulus was calculated as the slope of the linear region of the stress–strain curves (corresponding to 0–20% strain). Each experiment was carried out in triplicate.

### 4.7. Rheological Analyses of HA-MA Hydrogels

Rheological analyses of the HA-MA hydrogels were performed on an MCR302 Rheometer (Anton Para GmbH, Graz, Austria) in a 25 mm plate–plate configuration. After the complete gelation of each specimen, the limit of the linear viscoelastic region (LVE) was studied at room temperature through amplitude sweep tests in the γ range of 0.1–10%. Evaluation of the oscillatory responses (G′—elastic modulus and G″—loss/viscous modulus) was conducted at low strain values (γ = 0.1%) over the frequency range of 0.08–8 Hz, according to the literature [83,84].

### 4.8. Scanning Electron Microscopy (SEM) and Pore Dimensional Analysis of HA-MA Hydrogels

The morphological characterisation of the microstructures and pore sizes of the different HA-MA hydrogels were characterised using a Zeiss Gemini 300 (Jena, Germany) field emission scanning electron microscopy (20 kV). Hydrogels were sputter-coated with gold (CCU-010 LV, Safematic GmbH, Zizers, Switzerland), and multiple micrographs were imaged at 100× magnification (V = 2 kV, Wd~9 mm) from selected regions. The microstructural features of the 3D hydrogel network were analysed using the free software (Fiji is just) ImageJ 1.53t (National Institutes of Health, Bethesda, MD, USA) and Origin. First, the images were thresholded using the percentile method and then segmented to individuate the pores. The average values of the area, circularity, number of pores, Feret diameter (the longest distance between any two points along the selection boundary) and aspect ratio (ratio of major/minor axis of pores) of the hydrogels were quantified from three independent sets of images for each hydrogel. The microstructural features were calculated as follows:

(1) D=2×√(A/π)

(2) C=(4π×A)/P^2

where D is the equivalent diameter, C is the circularity, A is the area and P is the perimeter. The circularity (C) is defined as the degree to which the particle is similar to a circle, taking into consideration the smoothness of the perimeter. This means that the circularity is a measurement of both the particle form and roughness. Thus, the further away from a perfectly round, smooth circle a particle becomes, the lower the circularity value. Pores with circular shapes have an aspect ratio of 1.

### 4.9. Protein Absorption Measurement

Cell survival is strictly related to the capacity of a substrate to promote nutrient exchange. To analyse the protein uptake capacity, the hydrogel was immersed in an albumin solution (1 mg/mL) for 24 h. Following incubation, the protein concentration was measured using the DC Protein Assay Kit (Bio-Rad, Hercules, CA, USA) according to the manufacturer’s instructions, using a Glomax Discovery microplate reader. All experiments were performed in triplicate.

### 4.10. Cell Culture and Spheroid Formation

Human malignant glioma cells U251-MG were cultured in DMEM supplemented with 10% fetal bovine serum, 2 mM L-glutamine, 100 mg/mL streptomycin and 100 U/mL penicillin (Gibco BRL Life Technologies Inc., Grand Island, NY, USA) at 37 °C in a humidified atmosphere with 5% CO_2_. Cells were plated at an appropriate density according to each experimental setting. To generate U251-MG spheroids, the hanging drop cell culture protocol was used [85]. In brief, 20 μL drops with 1 × 10^3^ cells each were deposited so that they were sufficiently spaced on the internal surface of a lid from a 100 mm × 20 mm Petri dish. Thus, the lid was inverted onto the plate bottom filled with 5 mL of PBS and incubated at 37 °C in a humidified atmosphere with 5% CO_2_. After 72 h of incubation, the formed spheroids were carefully collected using a 200 mL pipette. Before seeding, the HA-MA hydrogels were sterilised through imbibition in 70% ethanol for 15 min, and subsequently washed with sterile MQ water and exposed to a cell hood UV lamp for 30 min. For 2D seeding, the spheroids were distributed on the top surface of the hydrogel. For 3D seeding, the spheroids were dispensed inside the hydrogel by mechanically penetrating the surface of the substrate and releasing spheroids inside via several injections. A concentration of approximately 20 spheroids/mL of hydrogel was used for each hydrogel.

### 4.11. Metabolic Activity Assay

The metabolic activity was evaluated using MTT assays. Hydrogels were prepared in 96-well plates in a volume of 100 µL, with a seeding of about 6 spheroids per hydrogel. For the sample of day 7, the medium was replaced on day 3. After 1, 3 and 7 days the medium was discarded and 0.5 mg/mL solution of MTT in DMEM was added and cells were incubated at 37 °C for 2 h. The supernatant was removed from each well and the formazan crystals were solubilised in 100 µL of isopropanol with 1% HCl. After 1 h, the optical density (OD) was measured at 560 nm, with a reference at 690 nm, using a microplate reader (Glomax Discovery). The results are shown as the percent viability relative to day 1, which was considered to be 100%.

### 4.12. Live/Dead Staining for Fluorescence Imaging

Cell viability was assessed using a LIVE/DEAD double-staining kit (04511-1KT-F, Sigma-Aldrich, St. Louis, MO, USA). In brief, cells were incubated in a cell culture medium supplemented with 0.25 μM of calcein (to label live cells) and 0.25 μM of propidium iodide (to label membrane-damaged or dead cells) for 30 min at 37 °C. After staining, the culture medium was replaced, and cells were imaged with a confocal laser scanning microscope (Olympus FV10i) with a 10× air objective. The total fluorescence was calculated using (Fiji is just) ImageJ 1.53t (National Institutes of Health, Bethesda, MD, USA). A region of interest was drawn around each cell and the total corrected fluorescence was calculated as follows: corrected total cell fluorescence (CTCF) = integrated density—(area of selected cell X mean fluorescence of background readings). The number of live cells present over time was normalised to day 1 and presented.

### 4.13. Morphological Analysis of Spheroids

To quantify the morphological parameters, spheroids were imaged after 1, 3 and 7 days to measure the spheroids’ area and estimate their circularity. Spheroids’ morphology was examined by acquiring phase contrast images on an Olympus IX73 inverted microscope, equipped with a QImaging OptiMOS sCMOS camera (Crisel, QImaging, Surrey, BC, Canada) and a stage-mounted incubator with CO_2_ and temperature control (H201; Okolab, Pozzuoli, Italy) using 10× magnification (Plan N, NA = 0.25, Ph1). Images from three independent experiments were processed using the particle measurement feature within (Fiji is just) ImageJ 1.53t (National Institutes of Health, Bethesda, MD, USA) to obtain the spread area and circularity of single spheroids. The circularity of spheroids was calculated using 4π (area/perimeter^2). Values of 1.0 designate a perfect circle, and values near zero are an indication of a more elongated morphology of cells.

### 4.14. Immunofluorescence

For the immunofluorescence analysis, the spheroids seeded on hydrogels were fixed after 1, 3 and 7 days using 4% paraformaldehyde in PBS for 30 min at room temperature before being permeabilised with 0.2% (*v*/*v*) Triton X-100 in PBS for 15 min and blocked with PBS containing 5% BSA. Cells were then incubated with Ki-67 (1:500, PA5-19462, Invitrogen, Schwerte, Germany) and phalloidin-TRITC (0.1 µg/mL, Sigma-Aldrich, Steinheim, Germany) for F-actin labelling in a blocking buffer. Fluorescent dye (DYE-Light)-conjugated secondary antibodies against rabbit IgG were used at a dilution of 1:1000 for 1 h at 37 °C in a blocking buffer. After washing in PBS, the samples were incubated with a DAPI (Sigma-Aldrich, Steinheim, Germany) solution in PBS (5 µg/mL) for 30 min. Qualitative images of spheroids were acquired using a confocal (Nikon, Tokyo, Japan) microscopy system equipped with 20× (UPlanFLN, NA 1.30, oil) and 60× (UPlanSApo, NA 1.35, oil) lenses.

### 4.15. Statistical Analyses

Statistical analyses were performed with GraphPad Prism 9.0 (GraphPad Software, Inc., San Diego, CA, USA). One-way analysis or two-way analysis of variance (ANOVA) and Sidak Tukey’s multiple-comparison post hoc test were used. Differences were considered statistically significant when *p* ≤ 0.05.

## Data Availability

Not applicable.

## Supplementary Materials

The following supporting information can be downloaded from https://www.mdpi.com/article/10.3390/gels9100801/s1. Table S1: Synthetic details of the different syntheses for modification of HA with methacrylate groups. Scheme S1: Scheme of experimental workflow. Scheme S2: Preparation of HA-MA hydrogels: details of photopolymerisation conditions using LAP. Scheme S3: Preparation of HA-MA hydrogels: details of photopolymerisation conditions using Irgacure. Figure S1: Representative ^1^H-NMR spectrum of HA-MA with low and high DMs. Figure S2: Representative FT-IR spectra of HA (red line) and HA-MA (blue line) comparing the chemical structure and interactions. Figure S3: Frequency sweeps of hydrogels exposed to different wavelengths and with different photoinitiators. Figure S4: Amplitude sweeps of hydrogels exposed to different wavelengths and with different photoinitiators. Figure S5: Quantification of hydrogel morphology from the SEM images. Figure S6: Quantification of protein absorbance on HA-MA hydrogels.
