# Supplementary material for: Modulation of Methacrylated Hyaluronic Acid Hydrogels Enables Their Use as 3D Cultured Model
Source: Gels. 2023 Oct 5;9(10):801. doi: 10.3390/gels9100801 (PMC10606912; doi:10.3390/gels9100801)
Supplement: Supplementary file 1 [file gels-09-00801-s001.zip › gels-2597437-supplementary.pdf]

gels

Supplementary Materials

Modulation of Methacrylated Hyaluronic Acid Hydrogels Enables Their Use as 3D Cultured Model

Ornella Ursini [1,*,†], Maddalena Grieco [2,†], Carla Sappino [3], Agostina Lina Capodilupo [2], Sara Maria Giannitelli [4], Emanuele Mauri [5,6], Alessio Bucciarelli [2], Chiara Coricciati [2,7], Valeria de Turris [8], Giuseppe Gigli [2,7], Lorenzo Moroni [2,9] and Barbara Cortese [1,*]

Table S1. Synthetic details of the different syntheses for modification of HA with methacrylate groups.

HA (g)	H_2O (mL)	Sodium Hyaluronate Solution % (w/v)	Ratio H_2O /DMF	DMF (mL)	Molar Ratio MA/HA	Degree of Methacrylation (DM)
2.93	150	~ 2	3:2	100	3.6	97- 98 %
1.00	100	1	3:2	66.6	2.9	24.7%
2.00	200	1	3:2	133	2.9	24.6%

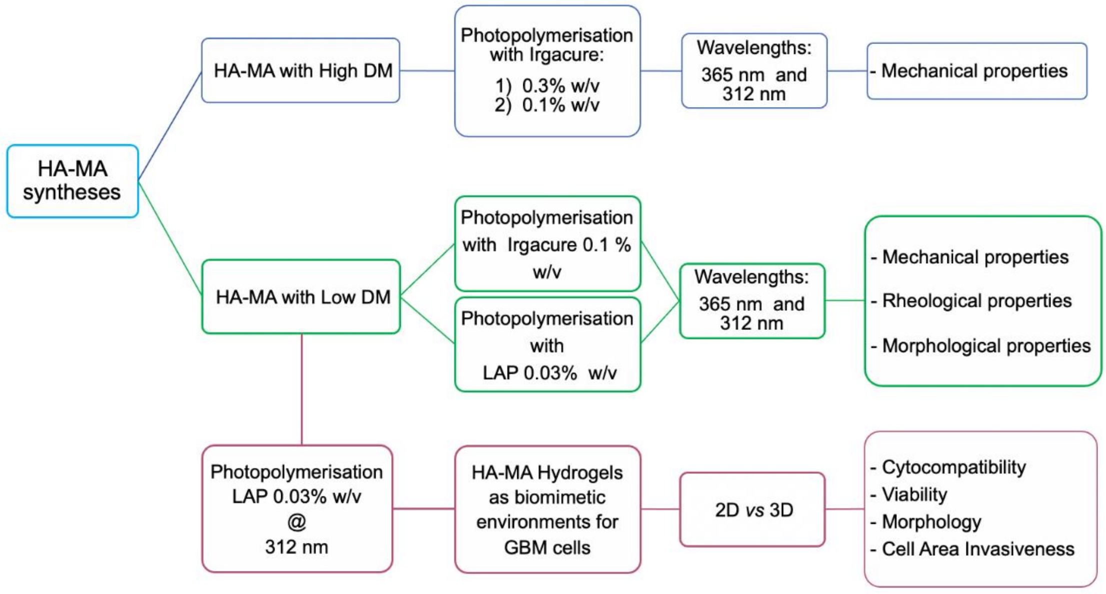

Scheme 1. Scheme of experimental workflow.

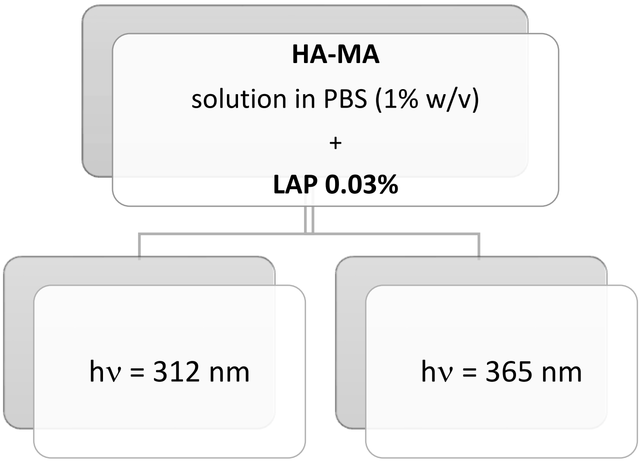

Scheme 2. Preparation of HA-MA hydrogels: details of photopolymerisation conditions. Graph of the different conditions used to obtain HA-MA hydrogels using LAP as a photocatalyst. The UV intensity of irradiation of the 312 nm source was equal to 1.5 mW/cm^2. The UV intensity of irradiation of the 365 nm source was equal to 0.7 mW/cm^2. Irradiation time was three minutes.

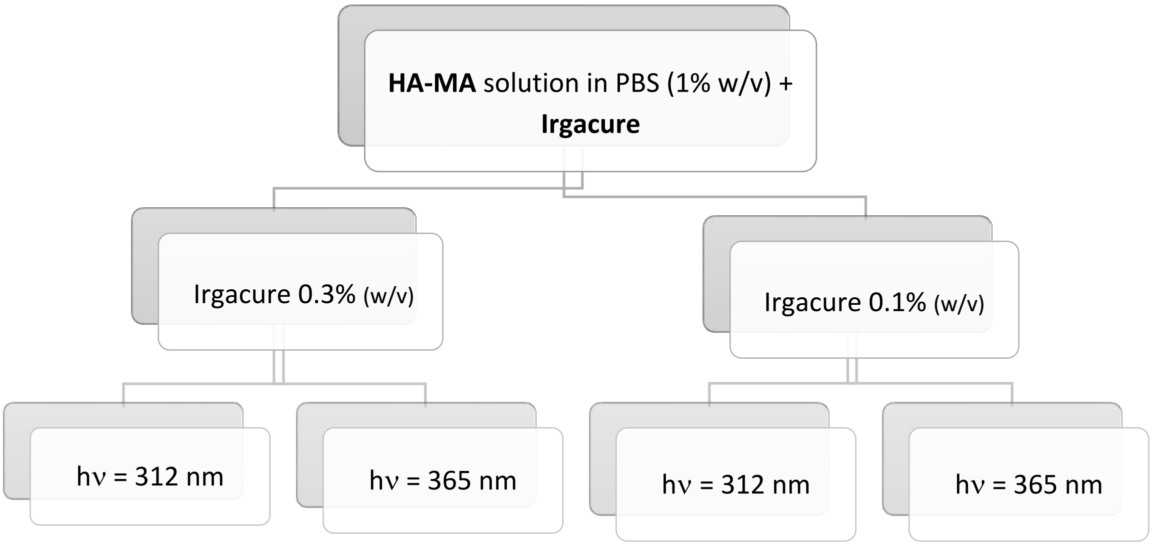

Scheme 3. Preparation of HA-MA hydrogels: details of photo-polymerisation conditions. Graph of the different conditions used to obtain HA-MA hydrogel using different concentrations of Irgacure as a photocatalyst. The UV intensity of irradiation of the 312 nm source was equal to 1.5 mW/cm^2. The UV intensity of irradiation of the 365 nm source was equal to 0.7 mW/cm^2. Irradiation time was three minutes.

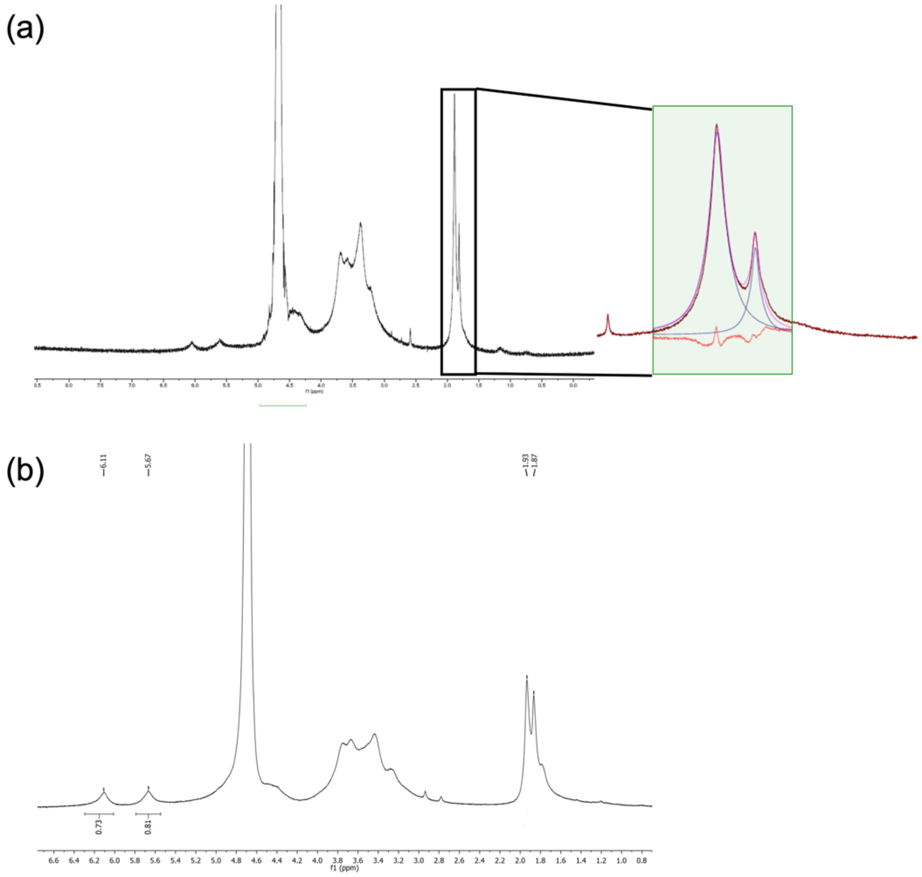

Figure S1. Representative ^{1}H-NMR spectrum of HA-MA. (a) ^{1}H-NMR spectrum with low DM (24%). Peaks correspond to the methyl protons in the MA residues linked and in HA acetamide groups. The inset in the figure shows the enlargement of 1H-NMR spectrum in the range of 2.0-1.6 ppm, which was used to evaluate the integrated intensities of the methacrylate protons. The DM was calculated by comparing the integral related to the methyl group belonging to HA residues (δ= 1.88 ppm) with the integral of the signal of the CH_3 belonging to MA (δ= 1.81 ppm). (b) ^{1}H-NMR spectrum with high DM (~ 98.0 %).

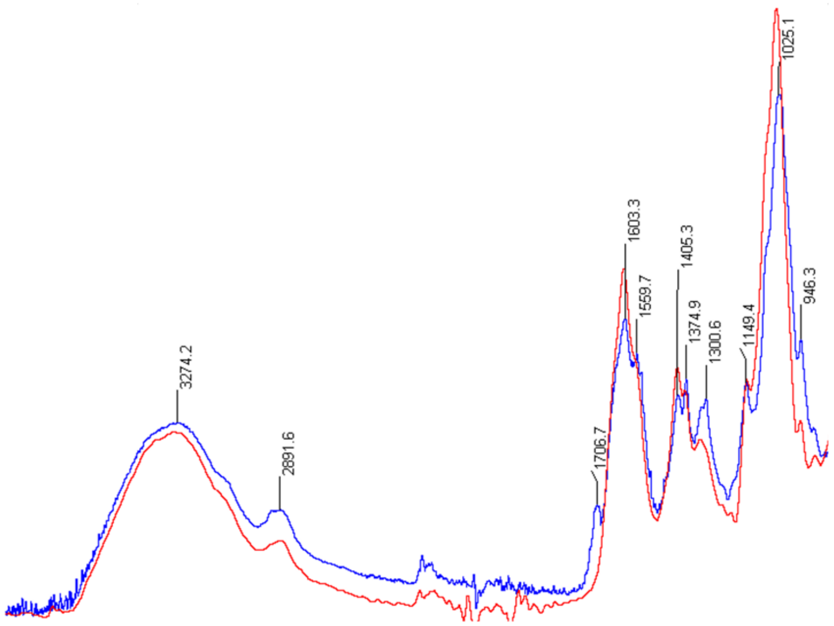

Figure S2. Representative FT-IR spectra of HA (red line) and HA-MA (blue line) comparing the chemical structures and interactions. New bands at 1706 cm^{-1} and 1300 cm^{-1} are clearly visible and were ascribed to the stretching of C=O groups (ν_{as} COO) and the scissoring of –C=C-H respectively. The band at 946 cm^{-1} was associated with the wagging of –C=C-H. The intensity of this band increased when methacrylate residues were linked to HA. .

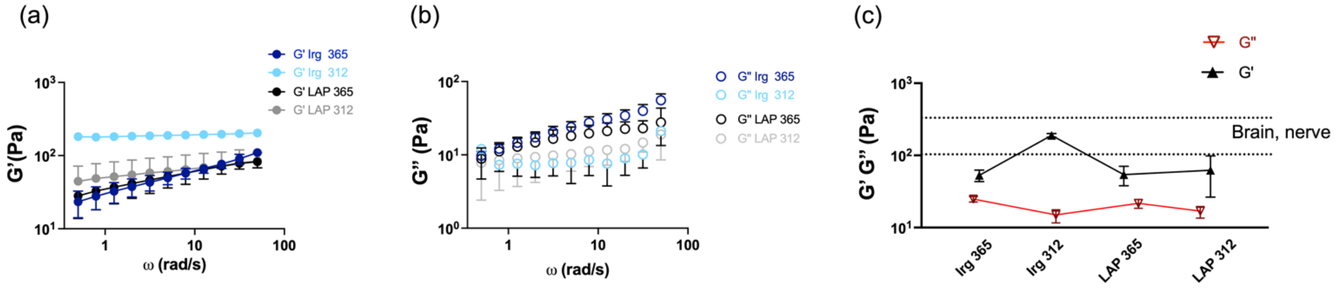

Figure S3. Frequency sweeps of hydrogels exposed to different wavelengths and with different photoinitiators showing (a) the storage moduli and (b) the loss moduli. (c) Storage (in black) and loss (in red) moduli of the HA-MA hydrogels at a frequency of 6.28 rad/s, with different photoinitiators and exposed to the two wavelengths, showing a storage modulus that remained close to the range of values of brain tissue and nerves (represented by the black dashed lines).

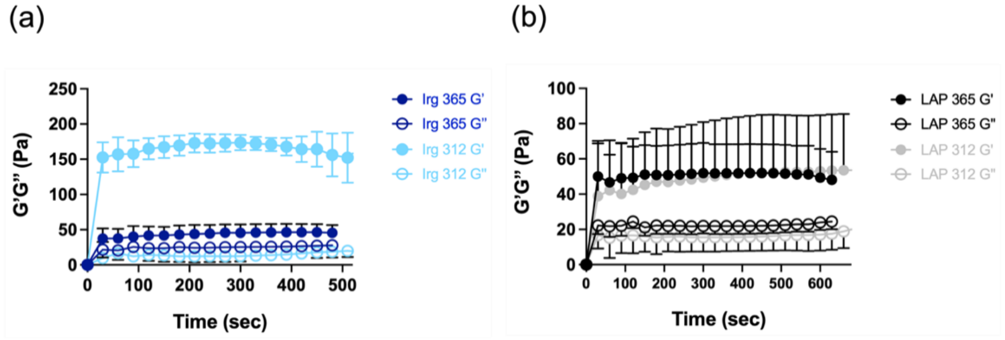

Figure S4. Amplitude sweeps of hydrogels exposed to different wavelengths and with different photoinitiators: (a) Irgacure and (b) LAP.

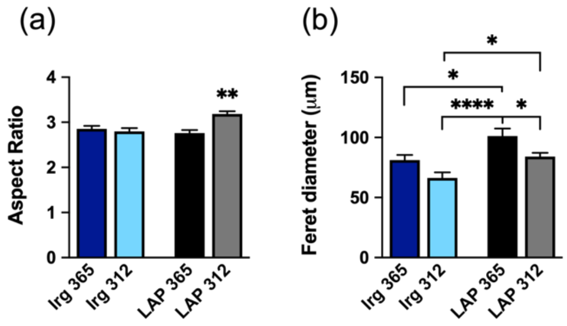

Figure S5. Quantification of hydrogel morphology from the SEM images: (a) aspect ratio and (b) Feret diameter. * indicates statistically significant difference using a one-way ANOVA with * $p < 0.5$, ** $p < 0.01$, *** $p < 0.001$ and **** $p < 0.0001$.

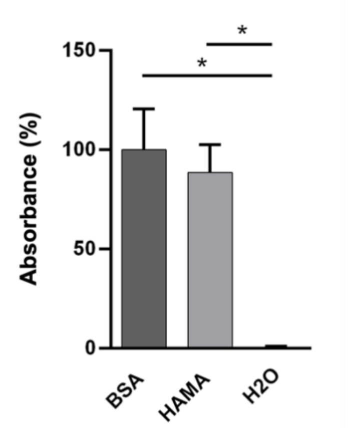

Figure S6. Quantification of protein absorbance on HA-MA hydrogels (with DM of 24.6% cross-linked with LAP at 312 nm), which was carried out using a DC Protein Assay Kit, as described in the Materials and Methods section. The results are shown as a percent relative to BSA, which was considered to be 100%. The values are the mean ± s.e.m. of three independent experiments. * indicates statistically significant difference using one-way ANOVA followed by Holm–Sidak post-hoc analysis; * $p<0.05$.
